# Validity of an FFQ to measure nutrient and food intakes in Tanzania

**DOI:** 10.1017/S1368980018000848

**Published:** 2018-04-16

**Authors:** Rachel M Zack, Kahema Irema, Patrick Kazonda, Germana H Leyna, Enju Liu, Susan Gilbert, Zohra Lukmanji, Donna Spiegelman, Wafaie Fawzi, Marina Njelekela, Japhet Killewo, Goodarz Danaei

**Affiliations:** 1 Department of Epidemiology, Harvard T.H. Chan School of Public Health, Kresge Building, Room 911, 677 Huntington Avenue, Boston, MA 02115, USA; 2 School of Public Health, Muhimbili University of Health and Allied Sciences, Dar es Salaam, United Republic of Tanzania; 3 Department of Global Health and Population, Harvard T.H. Chan School of Public Health, Boston, MA, USA; 4 Independent nutrition/dietetic consultant and consultant dietitian affiliated with Tumaini Comprehensive Infirmary, Dar es Salaam, United Republic of Tanzania; 5 Department of Biostatistics, Harvard T.H. Chan School of Public Health, Boston, MA, USA; 6 Department of Nutrition, Harvard T.H. Chan School of Public Health, Boston, MA, USA; 7 Department of Physiology, Muhimbili University of Health and Allied Sciences, Dar es Salaam, United Republic of Tanzania

**Keywords:** FFQ, Validation, Nutritional epidemiology, Tanzania, Sub-Saharan Africa

## Abstract

**Objective:**

FFQ are often used to estimate food and nutrient intakes to rank individuals by their level of intake. We evaluated the relative validity of a semi-quantitative FFQ created for use in Tanzania by comparing it with two 24 h diet recalls.

**Design:**

We measured relative validity of the FFQ with deattenuated energy-adjusted rank correlations for nutrients, deattenuated rank correlations for food groups, and performed a cross-classification analysis of energy-adjusted nutrient quartiles using percentage of agreement and Bland–Altman analysis.

**Setting:**

Interviews were conducted in 2014 in participants’ homes in Ukonga, Dar es Salaam, Tanzania.

**Subjects:**

We surveyed 317 adults aged 40 years or older from the general public.

**Results:**

Deattenuated energy-adjusted rank correlation coefficients of nutrients ranged from −0·03 for riboflavin to 0·41 for percentage of energy from carbohydrates, with a median correlation of 0·21. Coefficients for food groups ranged from 0·00 for root vegetables to 0·51 for alcohol, with a median of 0·35. Relative to the average of the two 24 h diet recalls, the FFQ overestimated energy intake and intakes of all nutrients and food groups, other than tea, with ratios among nutrients ranging from 1·34 for SFA to 7·08 for vitamin A; and among food groups from 0·92 for tea to 9·00 for fruit. The percentage of participants classified into the same nutrient intake quartile ranged from 23 % for SFA to 32 % for both niacin and pantothenic acid, with a median of 28 %.

**Conclusions:**

The FFQ performed moderately well in urban Tanzanian adults.

Non-communicable diseases are a growing concern in sub-Saharan Africa (SSA). In this region, the number of cardiovascular deaths has nearly doubled from 1990 to 2015^(^ ^1^ ^)^, the prevalence of diabetes has more than doubled from 1980 to 2014^(^ ^2^ ^)^ and the prevalence of hypertension and obesity are both increasing^(^ ^3^ ^,^ ^4^ ^)^. In rural SSA, the proportion of adult deaths due to non-communicable diseases has increased: from 16 % in 2003 to 24 % in 2007 in Tanzania^(^ ^5^ ^)^ and from 35 % in 2003 to 45 % in 2010 in Kenya^(^ ^6^ ^)^.

One of the potentially modifiable risk factors for non-communicable diseases is diet, which is well documented to affect the risk for many cardiometabolic diseases and cancers^(^ ^7^ ^–^ ^10^ ^)^. In SSA, although data remain sparse, it is clear that access to food is increasing but diet quality is worsening. Data from the FAO show that per capita energy intake has increased over the past 30 years^(^ ^11^ ^)^, snack food and soft drink importation to the fifteen countries that make up the Southern African Development Community has quadrupled over the past two decades^(^ ^12^ ^)^, and overall dietary quality has worsened^(^ ^13^ ^)^. Reflecting the consequences of the worsening dietary situation in SSA, the Global Burden of Disease study estimates that the proportion of deaths attributable to unhealthy diet in Tanzania has increased from 5·8 % in 1990 to 9·3 % in 2015^(^ ^14^ ^,^ ^15^ ^)^.

As the burden of non-communicable diseases in SSA continues to grow, more research is needed to discover the causes of this rising burden and help governments develop prevention policies. Well-designed public health policies could influence diet through such means as education to inform and change food preferences, healthy food production, food subsidization and taxation^(^ ^16^ ^–^ ^19^ ^)^. However, obtaining reliable information on dietary intake can be challenging because validated tools for quantifying diet in SSA are limited. FFQ are the standard tool used to estimate dietary intake for diet–disease analyses in large cohorts^(^ ^20^ ^)^. Such questionnaires, which were developed in the 1980s, have been widely used by researchers in the USA and Europe. However, as the validity of FFQ is sensitive to cultural and regional factors^(^ ^20^ ^–^ ^22^ ^)^, FFQ need to be validated before being extended for use outside the USA and Europe, such as in SSA where diets, availability of foods and dishes, portion sizes, numeracy, literacy and customs differ from those in the USA and Europe^(^ ^23^ ^,^ ^24^ ^)^. Within SSA, full-length FFQ intended to measure the entire diet have been validated for use in Mali, South Africa and Botswana^(^ ^25^ ^–^ ^29^ ^)^. However, diets differ between SSA countries, and an FFQ has not yet been validated for use in Tanzania. A previous validation of a Tanzanian FFQ against two 24 h diet recalls provided only correlation coefficients and *P* values for six broad food groups (fruits, cereals, beverages, vegetables, animal products, fats) based on data from fifty women^(^ ^30^ ^)^. Here, we report the validity of estimated intakes of energy, twenty-five nutrients and thirteen food groups as assessed by our FFQ compared with two repeated 24 h diet recalls among adults in Dar es Salaam, Tanzania.

## Methods

### Sampling design and participants

The Dar es Salaam Health and Demographic Surveillance System (HDSS) was initiated in 2011 and collected demographic data on all individuals living in Ukonga, a peri-urban ward of Dar es Salaam^(^ ^31^ ^)^. For the Dar es Salaam Urban Cohort Hypertension Study (DUCS-HTN), we attempted to contact everyone registered in the HDSS, aged 40 years or older, from two randomly selected neighbourhoods of the seven neighbourhoods in the HDSS (*n* 4896). Additional information on the DUCS-HTN has been reported previously^(^ ^32^ ^)^. We randomly selected 1024 of these potential participants to contact for the DUCS-HTN dietary sub-study. We excluded participants who were pregnant or physically or mentally incapable of participating in the DUCS-HTN. For inclusion in the analytical sample, we required that participants complete an FFQ and two 24 h diet recalls, have fewer than 10 % of their FFQ items missing and that their daily energy intake from the FFQ be within a plausible range of 2092–20 920 kJ (500–5000 kcal).

The Institutional Review Board of the Harvard T.H. Chan School of Public Health and the Muhimbili University of Health and Allied Sciences Ethical Committee approved the study protocol. Written informed consent was obtained from all participants; or, if the participant was unable to sign, a witness signed on behalf of the participant.

### Dietary assessments

Trained interviewers conducted face-to-face interviews and physical examinations in participants’ homes from March to June 2014. The six interviewers who conducted dietary questionnaires had experience in public health data collection, but not in collecting FFQ or 24 h diet recalls. The interviewers therefore received one full day of training and written instructions on how to conduct the FFQ and 24 h diet recall. A nutritionist and epidemiologists conducted the dietary data collection training. Interviewers had two days of field practice before commencing data collection, during which supervisors observed interviews and provided feedback to the interviewers to standardize the interview technique between the six interviewers. Interviewers administered the FFQ and the first 24 h diet recall at the first study visit. All interviews and examinations proceeded in the same order: lifestyle questionnaire, blood pressure measurements, FFQ, anthropometric measurements, 24 h diet recall, capillary blood measurements and finally provision of instructions for the 24 h urine collection. A second 24 h diet recall was repeated at the start of the next study visit, a minimum of three days later. The median number of days between the two 24 h diet recalls was 6 (interquartile range 4–15). Eighty-four per cent of participants completed both 24 h diet recalls during the workweek, 15 % completed one on the weekend and one during the week, and 2 % completed both during the weekend.

Interviewers verbally administered the 24 h diet recall to participants. To help standardize participants’ understanding of serving size, the interviewers showed participants plastic sample dishes as well as drawn images of portion sizes (see images in the online supplementary material) for the 24 h diet recalls and the FFQ.

Interviewers verbally administered the 179-food-item semi-quantitative FFQ with specified portion sizes and a recall period of 30 d. The FFQ was slightly modified, through the addition of food items, from the FFQ created by Lukmanji *et al*.^(^ ^33^ ^)^. Similar FFQ without published validation studies have previously been used in Tanzania^(^ ^34^ ^)^. There were nine options for frequency of consumption, with servings per day calculated from these frequencies as follows: ‘never consumed over past thirty days’ (0 servings/d), ‘1–3 servings per month’ (‘0·067 servings/d), ‘1 serving per week’ (0·143 serving/d), ‘2–4 servings per week’ (0·429 servings/d), ‘5–6 servings per week’ (0·786 servings/d), ‘1 serving per day’ (‘1 serving/d), ‘2–3 servings per day’ (2·5 servings/d), ‘4–5 servings per day’ (4·5 servings/d) and ‘6+ servings per day’ (6 servings/d). Participants selected one frequency of consumption for each of the 179 items in the FFQ. Portion sizes were provided for each food item (see FFQ in the online supplementary material); however, participants could report that they consumed a fraction of a serving size (e.g. ½ or ¼).

For both the 24 h diet recall and FFQ data, nutrient information for individual foods was taken from the 2008 Tanzania Food Composition Tables^(^ ^35^ ^)^. These food composition tables were based primarily on data from WorldFood Dietary Assessment System, with some information also taken from the US Department of Agriculture’s food composition database and the South Africa food composition tables. Chemical analysis of the nutrient composition of a small number of foods (mainly maize and some leafy greens) was conducted at Sokoine University of Agriculture. The Tanzanian Food Composition Tables include nutrient composition of mixed dishes. The authors of the Tanzanian Food Composition Tables collected sample recipes from a variety of sources (e.g. street food vendors, student canteen, recipe books and websites). The nutrient composition of the recipe was then estimated from the nutrient composition of the individual ingredients. These tables provide information on the energy and nutrient contents and grams per serving size of over 400 foods and dishes commonly consumed in Tanzania. Nutrient intakes for each individual included contributions from foods but not supplements.

### Statistical analysis

Descriptive statistics (means and sd) were calculated for energy, nutrient and food group intakes as estimated by the FFQ and the average of the two 24 h diet recalls. Foods were categorized into thirteen groups (see online supplementary material, Supplemental Table 1) for food group analyses. Nutrient intakes were adjusted for total energy intake using the residual method^(^ ^36^ ^)^. We also adjusted for total energy intake when analysing macronutrient intakes by calculating macronutrient intakes as a percentage of total energy intake (i.e. the nutrient density method). Fats, carbohydrates and protein were separately adjusted for total energy intake using the nutrient density method.

**Table 1:** Basic characteristics of Dar es Salaam Urban Cohort Hypertension Study (DUCS-HTN) participants, 2014 (*n* 317)

Characteristic	%
Age (years)	
Median	52
IQR	45–60
Age group	
40–44 years	21
45–49 years	21
50–54 years	20
55–59 years	12
60–64 years	11
65–69 years	7
≥70 years	9
Male	39
Religion	
Muslim	51
Christian	49
Education	
None	12
At least some primary	64
At least some secondary	25
BMI (kg/m^2^)	
Median	27·2
IQR	23·0–31·8
BMI category	
Underweight (<18·5 kg/m^2^)	6
Normal weight (18·5–24·9 kg/m^2^)	29
Overweight (25·0–29·9 kg/m^2^)	31
Obese (≥30·0 kg/m^2^)	34

Rank correlation coefficients and 95 % CI were calculated to evaluate the strength of the associations between nutrient and food group intakes derived from the FFQ *v.* the average of the two 24 h diet recalls^(^ ^37^ ^)^. To compare nutrient intakes as measured by the two different dietary assessment methods, we calculated unadjusted, energy-adjusted and deattenuated energy-adjusted correlation coefficients. To compare food group intakes as measured by the two different dietary assessment methods, we calculated unadjusted and deattenuated correlation coefficients. Deattenuated correlations were used to adjust for random within-person variation across the 24 h diet recalls^(^ ^37^ ^)^. We also calculated intraclass correlations between nutrient and food group intakes as measured by the FFQ and average of the two 24 h diet recalls.

We examined the ability of the two dietary questionnaires to categorize participants into the same energy-adjusted nutrient intake quartiles. The proportions of individuals who were classified correctly within the same quartile, within the same or adjacent quartile, and in opposite quartiles (lowest quartile according to one dietary questionnaire and highest quartile according to the other) were determined. Kappa statistics with linear weights were calculated to further quantify the agreement between energy-adjusted nutrient intake quartiles as measured by the FFQ and average of two 24 h diet recalls^(^ ^38^ ^,^ ^39^ ^)^.

We examined systematic differences in macronutrient intakes as measured by the FFQ and 24 h diet recalls by creating Bland–Altman plots of energy intake, percentage of energy from fat, percentage of energy from protein and percentage of energy from carbohydrates^(^ ^40^ ^)^. The plots were created by plotting the difference in nutrient intake from the two dietary intake measurement methods against the mean of the nutrient intake from the two dietary intake measurement methods. A relationship between the difference and the mean values indicates systematic bias (e.g. the FFQ tends to overestimate nutrient intake more for those with higher total energy intakes). Limits of agreement (mean(nutrient_FFQ_ – nutrient_24hDR_)±1·96×sd(nutrient_FFQ_ – nutrient_24hDR_)) were calculated and plotted.

Analyses were performed using the statistical software package SAS version 9.3. Figures were created using R version 2.15.3.

## Results

**Fig. 1:**
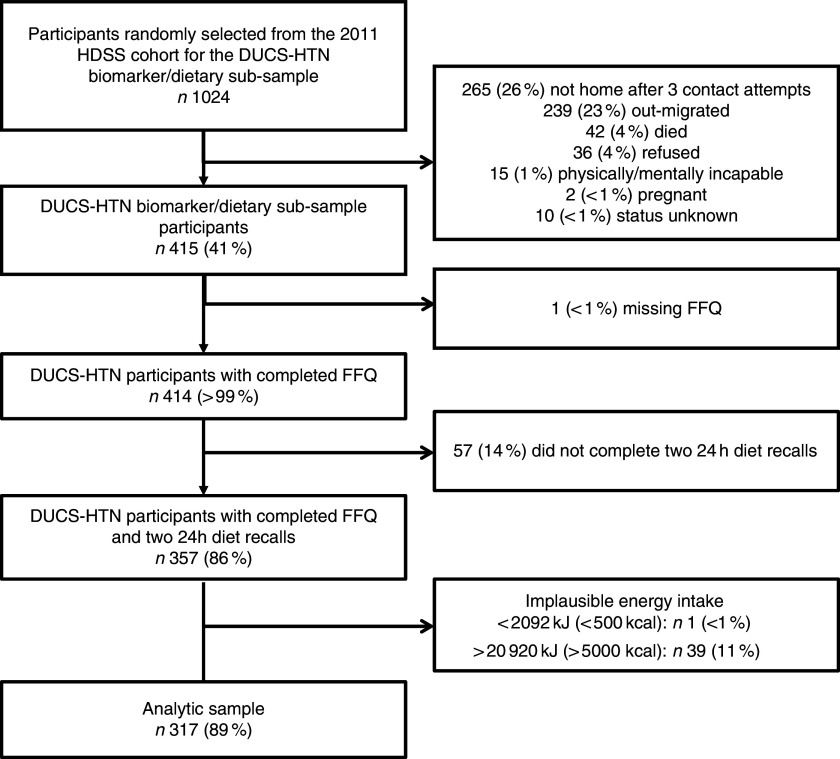
Flowchart of study participation and data completeness in the Dar es Salaam Urban Cohort Hypertension Study (DUCS-HTN), 2014; which was a sub-sample of participants from the Dar es Salaam Health and Demographic Surveillance System (HDSS), 2011

Among the 1024 participants whom we attempted to contact for the DUCS-HTN dietary sub-study, 265 were not home after three contact attempts, 239 had out-migrated, forty-two had died, fifteen were mentally or physically incapable of participating, thirty-six refused to participate, two were pregnant and ten did not enroll for unknown reasons (Fig. 1), resulting in a sample of 415 participants. Of these individuals, 414 completed the FFQ and 357 also completed the two 24 h diet recalls. None of the FFQ had missing data on more than 10 % of FFQ food items. We excluded one participant who, according to the FFQ, consumed <2092 kJ/d (<500 kcal/d), as well as thirty-nine participants who consumed >20 920 kJ/d (>5000 kcal/d), which led to an analytical sample of 317 participants. Among these, the median age was 52 (interquartile range 45–60) years, 61 % of participants were female, 65 % were overweight or obese, and 75 % had less than secondary education (Table 1).

The mean daily energy, nutrient and food intakes estimated by the FFQ were higher than those estimated by the average of the two 24 h diet recalls (Table 2). This was true for all nutrients and all foods, other than tea. Total energy intake as estimated by the FFQ was 1·68 times that estimated by the average of the two 24 h diet recalls: mean of 10 874 (sd 4310) kJ (2599 (sd 1030) kcal) according to the FFQ and 6468 (sd 1803) kJ (1546 (sd 431) kcal) according to the average of the two 24 h diet recalls. The ratio of nutrient intake comparing FFQ estimates with 24 h diet recall estimates ranged from 1·34 for SFA to 7·08 for vitamin A. Among food groups, the ratio of consumption comparing FFQ estimates with 24 h diet recall estimates ranged from 0·92 for tea to 9·00 for fruits.

**Table 2:** Daily food and nutrient intakes estimated by the FFQ and two 24 h diet recalls in the Dar es Salaam Urban Cohort Hypertension Study (DUCS-HTN), 2014 (*n* 317)

	FFQ		24 h diet recall average	
Item (units/d)	Mean	sd	Mean	sd
Energy				
(kJ)	10 874	4310	6468	1803
(kcal)	2599	1030	1546	431
Macronutrients				
Carbohydrate (g)	361	145	237	65
Carbohydrate (% of energy)	67	8	73	7
Protein (g)	94	50	38	15
Protein (% of energy)	17	5	11	4
Fat (g)	91	42	52	22
Fat (% of energy)	17	4	16	5
SFA (g)	50	25	36	17
MUFA (g)	23	11	8	4
PUFA (g)	12	9	4	3
Cholesterol (mg)	185	124	51	46
Vitamins				
Vitamin A (μg RAE)	1762	1461	249	394
Niacin (mg)	18	9	8	3
Pantothenic acid (mg)	6	3	3	1
Thiamin (mg)	2	2	0·8	0·3
Riboflavin (mg)	3	2	0·9	1·1
Vitamin B_6_ (mg)	2	1	0·9	0·4
Vitamin B_12_ (μg)	6	7	2·0	2·7
Folate (μg)	471	229	136	65
Vitamin C (mg)	163	104	39	37
Vitamin D (μg)	6	6	1·5	2·9
Vitamin E (μg)	10	6	2·4	1·7
Minerals				
Ca (mg)	604	508	283	282
P (mg)	1271	678	677	262
Fe (mg)	19	16	7	5
Mg (mg)	546	361	230	104
Na (mg)	957	540	377	224
K (mg)	3800	1806	1271	513
Zn (mg)	9	5	5	2
Food groups (servings)				
Cereals	5·0	1·9	2·7	0·9
Legumes & nuts	0·8	0·8	0·5	0·6
Root vegetables	0·6	0·8	0·4	0·5
Vegetables (excluding roots)	3·8	2·7	0·7	0·7
Fruit	1·8	1·3	0·2	0·4
Unprocessed red meat	0·6	0·5	0·4	0·5
Fish	0·9	0·7	0·5	0·5
Chicken	0·2	0·3	0·1	0·3
Eggs	0·2	0·2	0·0	0·1
Dairy	0·6	0·7	0·1	0·3
Sugar-sweetened beverages	0·2	0·4	0·1	0·2
Alcohol	0·2	0·7	0·1	0·2
Tea	1·2	0·7	1·3	0·7

RAE, retinol activity equivalents.

The intraclass correlations between the FFQ and average of the two 24 h diet recalls for nutrients ranged from 0·09 for vitamin A to 0·38 for total energy and for food groups it ranged from 0·12 for vegetables (excluding roots) to 0·58 for tea. Energy adjustment tended to reduce correlations and deattenuation tended to increase correlations (Table 3). The median deattenuated energy-adjusted correlation for nutrients was 0·21 and ranged from −0·03 for riboflavin to 0·41 for percentage of energy from carbohydrates. Macronutrients when measured as a percentage of total energy intake (i.e. the nutrient density method) had higher correlations than did macronutrients adjusted for energy using the residual method: 0·41 *v.* 0·25 for carbohydrates, 0·40 *v.* 0·22 for protein and 0·36 *v.* 0·15 for fat. The median deattenuated correlation for food groups was 0·35 and ranged from 0·00 for root vegetables to 0·51 for alcohol.

**Table 3:** Correlations and intraclass correlations (ICC) of daily intakes of nutrients and food groups as assessed with the average of two 24 h diet recalls and the FFQ in the Dar es Salaam Urban Cohort Hypertension Study (DUCS-HTN), 2014 (*n* 317)

	Rosner rank correlation^(^ ^37^ ^)^						
Item	Unadjusted	95 % CI	Energy-adjusted*	95 % CI	Deattenuated*	95 % CI	ICC†
Energy (kcal)	0·12	0·02, 0·22	–		0·16	0·02, 0·30	0·38
Nutrients (median)	0·15		0·12		0·21		0·21
Macronutrients (median)	0·22		0·16		0·26		0·20
Carbohydrate (g)	0·07	−0·04, 0·17	0·16	0·06, 0·26	0·25	0·09, 0·40	0·26
Carbohydrate (% of energy)	0·23	0·12, 0·32	–		0·41	0·24, 0·55	0·18
Protein (g)	0·20	0·10, 0·30	0·11	0·01, 0·22	0·22	0·03, 0·39	0·18
Protein (% of energy)	0·22	0·12, 0·32	–		0·40	0·24, 0·54	0·18
Fat (g)	0·21	0·10, 0·31	0·09	−0·02, 0·19	0·15	−0·03, 0·33	0·20
Fat (% of energy)	0·22	0·12, 0·32	–		0·36	0·19, 0·51	0·16
SFA (g)	0·18	0·08, 0·28	0·04	−0·07, 0·14	0·06	−0·12, 0·24	0·20
MUFA (g)	0·24	0·13, 0·33	0·26	0·16, 0·36	0·40	0·25, 0·53	0·26
PUFA (g)	0·15	0·04, 0·25	0·17	0·06, 0·27	0·26	0·10, 0·41	0·26
Cholesterol (mg)	0·26	0·16, 0·36	0·17	0·06, 0·27	0·26	0·10, 0·41	0·26
Vitamins (median)	0·11		0·12		0·19		0·25
Vitamin A (μg RAE)	0·09	−0·01, 0·20	0·05	−0·06, 0·15	0·11	−0·14, 0·35	0·09
Niacin (mg)	0·14	0·04, 0·24	0·14	0·03, 0·24	0·25	0·06, 0·42	0·18
Pantothenic acid (mg)	0·13	0·03, 0·23	0·19	0·09, 0·29	0·26	0·12, 0·39	0·36
Thiamin (mg)	−0·04	−0·15, 0·06	0·15	0·05, 0·25	0·24	0·08, 0·39	0·26
Riboflavin (mg)	0·16	0·05, 0·26	−0·02	−0·12, 0·09	−0·03	−0·19, 0·14	0·25
Vitamin B_6_ (mg)	0·10	−0·00, 0·21	0·13	0·02, 0·23	0·19	0·03, 0·33	0·29
Vitamin B_12_ (μg)	0·15	0·04, 0·25	0·08	−0·02, 0·19	0·14	−0·04, 0·31	0·21
Folate (μg)	0·00	−0·10, 0·11	0·08	−0·03, 0·18	0·12	−0·04, 0·28	0·25
Vitamin C (mg)	0·11	0·01, 0·22	0·12	0·01, 0·22	0·19	0·02, 0·34	0·25
Vitamin D (μg)	0·12	0·01, 0·22	0·02	−0·09, 0·12	0·03	−0·16, 0·22	0·18
Vitamin E (μg)	0·02	−0·08, 0·13	0·12	0·01, 0·22	0·21	0·03, 0·37	0·20
Minerals (median)	0·09		0·15		0·26		0·23
Ca (mg)	0·10	−0·01, 0·20	0·15	0·05, 0·26	0·26	0·09, 0·42	0·22
Fe (mg)	0·04	−0·06, 0·15	0·18	0·07, 0·28	0·27	0·12, 0·42	0·26
Mg (mg)	0·05	−0·06, 0·15	0·16	0·06, 0·27	0·26	0·10, 0·40	0·26
Na (mg)	0·20	0·10, 0·30	0·16	0·06, 0·26	0·25	0·09, 0·40	0·27
P (mg)	0·09	−0·01, 0·20	0·14	0·04, 0·24	0·26	0·08, 0·43	0·17
K (mg)	0·09	−0·02, 0·19	0·15	0·05, 0·25	0·25	0·08, 0·41	0·23
Zn (mg)	0·11	0·01, 0·21	0·12	0·02, 0·22	0·22	0·04, 0·40	0·17
Food groups (median)	0·21		–		0·35		0·25
Cereals	0·21	0·11, 0·31	–		0·29	0·15, 0·42	0·34
Legumes & nuts	0·01	−0·09, 0·12	–		0·02	−0·17, 0·21	0·18
Root vegetables	0·00	−0·11, 0·11	–		0·00	−0·16, 0·16	0·25
Vegetables (excluding roots)	0·01	−0·09, 0·12	–		0·02	−0·20, 0·25	0·12
Fruit	0·11	0·01, 0·21	–		0·16	0·01, 0·32	0·28
Unprocessed red meat	0·23	0·13, 0·33	–		0·47	0·33, 0·59	0·15
Fish	0·08	−0·02, 0·19	–		0·18	−0·03, 0·38	0·13
Chicken	0·22	0·12, 0·32	–		0·38	0·22, 0·52	0·20
Eggs	0·20	0·10, 0·30	–		0·40	0·23, 0·54	0·15
Dairy	0·25	0·14, 0·34	–		0·35	0·21, 0·48	0·32
Sugar-sweetened beverages	0·25	0·15, 0·35	–		0·37	0·22, 0·50	0·29
Alcohol	0·42	0·33, 0·51	–		0·51	0·40, 0·60	0·55
Tea	0·35	0·26, 0·44	–		0·41	0·30, 0·51	0·58

RAE, retinol activity equivalents.

* Nutrient intakes, but not food group intakes, were adjusted for total energy intake by the residual method.

† ICC for residual method energy-adjusted nutrients except for energy and ‘percentage of energy’ variables.

Cross-classification analyses of estimated nutrient intakes found low to moderate agreement (Table 4). The median proportion classified within the same quartile was 29 %, which is higher than the 25 % that is expected due to chance alone. The proportion correctly classified within the same quartile ranged from 23 % for SFA to 32 % for niacin and pantothenic acid. The proportion classified within the same or adjacent quartiles ranged from 60 % for riboflavin to 72 % for MUFA. The proportion classified into opposite quartiles (first quartile according to one questionnaire and fourth quartile according the other) ranged from 7 % for percentage of energy from protein to 13 % for total energy, cholesterol, vitamin A and riboflavin. The median proportion classified into opposite quartiles was 10 %, which is the same as what would be expected by chance. Weighted kappa values ranged from −0·03 for riboflavin to 0·14 for niacin and pantothenic acid.

**Table 4:** Cross-classification of energy-adjusted daily intakes of nutrients and food groups in quartiles as assessed with the average of two 24-h diet recall and the FFQ in the Dar es Salaam Urban Cohort Hypertension Study (DUCS-HTN), 2014 (*n* 317)

	Correctly classified (%)	Same or adjacent quartile (%)	Opposite quartiles (%)	Weighted kappa
Expected if randomly distributed	25	63	13	0·00
Energy (kcal)	28	68	13	0·08
Macronutrients				
Carbohydrate (g)	28	65	11	0·05
Carbohydrate (% of energy)	29	66	9	0·09
Protein (g)	28	66	8	0·09
Protein (% of energy)	31	64	7	0·11
Fat (g)	26	64	11	0·03
Fat (% of energy)	29	66	11	0·08
SFA (g)	23	60	11	−0·02
MUFA (g)	25	72	9	0·10
PUFA (g)	29	69	10	0·10
Cholesterol (mg)	26	66	13	0·04
Vitamins				
Vitamin A (μg RAE)	27	64	13	0·02
Niacin (mg)	32	69	9	0·14
Pantothenic acid (mg)	32	69	8	0·14
Thiamin (mg)	29	66	9	0·09
Riboflavin (mg)	26	60	13	−0·03
Vitamin B_6_ (mg)	29	69	9	0·11
Vitamin B_12_ (μg)	29	68	10	0·10
Folate (μg)	30	66	11	0·08
Vitamin C (mg)	25	64	9	0·04
Vitamin D (μg)	24	62	11	0·01
Vitamin E (μg)	30	64	12	0·06
Minerals				
Ca (mg)	31	68	9	0·12
Fe (mg)	31	68	10	0·11
Mg (mg)	31	68	12	0·09
Na (mg)	31	70	10	0·13
P (mg)	29	69	11	0·10
K (mg)	26	65	10	0·05
Zn (mg)	26	65	10	0·05
Median	28	66	10	0·07

RAE, retinol activity equivalents.

**Fig. 2:**
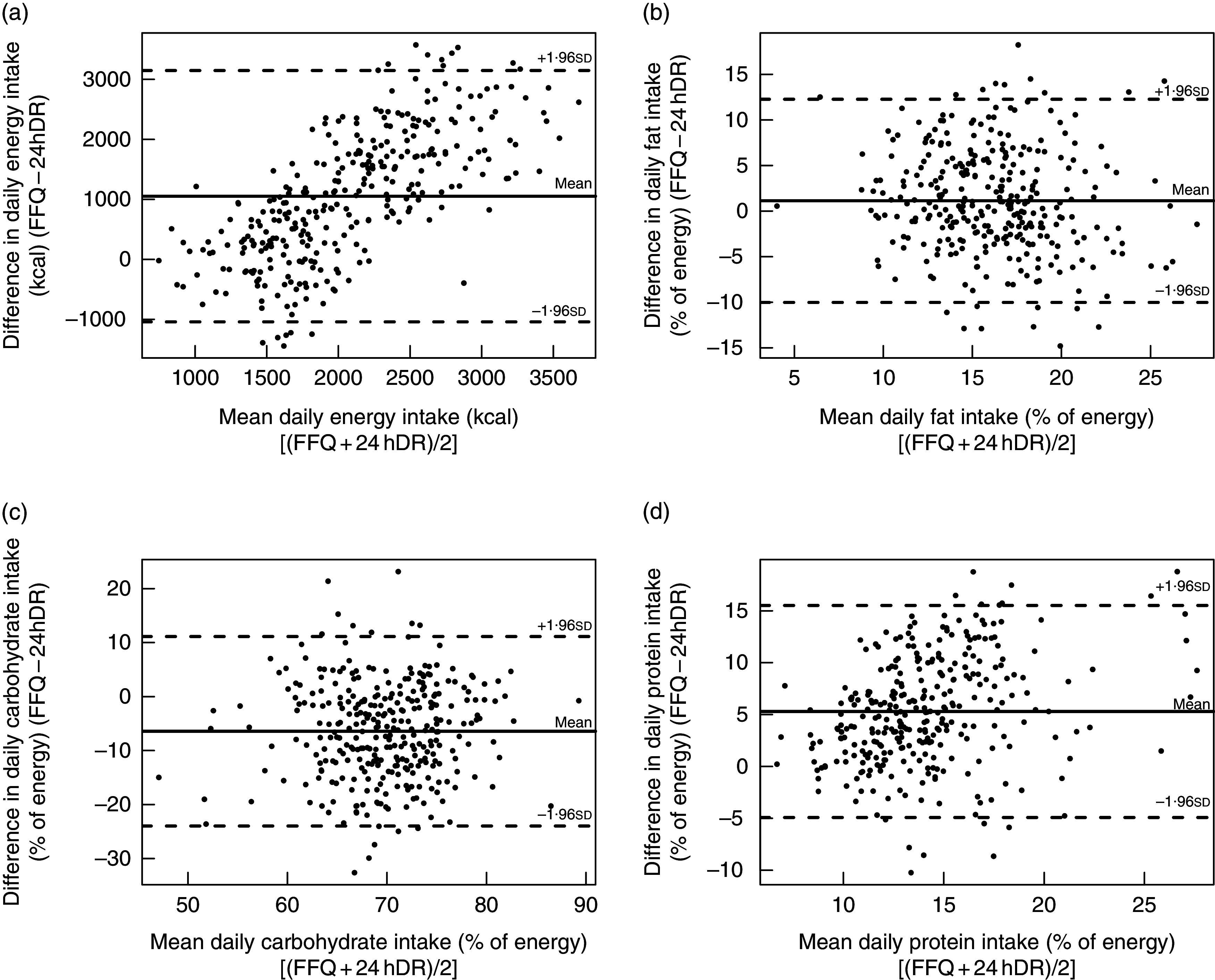
Bland–Altman plots to assess agreement between the FFQ and the average of two 24 h diet recalls (24 hDR) for (a) energy (to convert to kJ, multiply kcal values by 4·184), (b) fat (percentage of energy), (c) carbohydrates (percentage of energy) and (d) protein (percentage of energy) in the Dar es Salaam Urban Cohort Hypertension Study (DUCS-HTN), 2014 (*n* 317)

Bland–Altman plots of energy and percentage of energy from the three major macronutrients show moderate agreement (Fig. 2). The plots also show potential systemic bias in energy intake because the difference in energy intake between the FFQ and 24 h diet recalls is larger among participants with higher mean reported energy intake. However, systematic bias was not seen in percentage energy from fat, protein or carbohydrates. Ranges for limits of agreement were relatively wide, which indicates that there was wide variability in how the FFQ measured macronutrient intake relative to the average of the two 24 h diet recalls.

## Discussion

Our results indicate moderate validity of the Tanzanian FFQ when compared with two 24 h diet recalls in an urban adult population in Dar es Salaam. As has been observed in previous validation studies, we found that the FFQ generally overestimated intakes relative to the 24 h diet recalls^(^ ^25^ ^,^ ^29^ ^,^ ^41^ ^–^ ^44^ ^)^ and deattenuation tended to increase estimated correlation coefficients^(^ ^45^ ^)^. Coefficients comparing estimated nutrient intakes from the FFQ and two 24 h diet recalls were low to moderate. We found that the FFQ performed best for macronutrients when quantified as a percentage of energy intake, but less well for nutrients adjusted for energy intake using the residual method. The FFQ appeared to be a better measure of macronutrients and minerals than of vitamins. This may be because vitamin intake tends to vary greatly from day to day (as many vitamins are found in only a small selection of foods), whereas macronutrient intake remains relatively constant.

The validity of the FFQ to measure food group intakes was also moderate. However, we found that the FFQ has poor validity for measuring legume and nut intake, and vegetable (both root vegetables and other vegetables) intake. The poor validity of the FFQ for these food groups may be due to the difficulty of quantifying the intake of foods that tend to be a component of a dish, such as vegetables added to stews. This can be particularly challenging in Tanzania, where vegetables are often consumed in mixed dishes along with many other vegetables, and possibly meat or fish, rather than independently. Tanzanian meals are often composed of a starch (i.e. rice or stiff porridge (called *ugali* in Swahili)) and a stew, making it difficult for individuals to assess the quantity of each component of the food. An additional challenge in Tanzania and elsewhere in SSA is that food is often consumed communally, from a shared household dish rather than from individual plates, which complicates estimating portion size, as well as portion content. To account for this, we included ‘handful’ (*ujazo wa kiganja cha mkono* in Swahili) as a serving size option in the FFQ.

Previous FFQ validation studies have similarly found that correlations tend to be lower for vegetables than other food groups^(^ ^22^ ^,^ ^25^ ^,^ ^44^ ^,^ ^46^ ^–^ ^48^ ^)^. A cross-check question on the daily number of servings of vegetables could be added to future versions of the FFQ to help correct for over-reporting of vegetable intake^(^ ^22^ ^)^.

Our results are similar to those from previous FFQ validations that have been conducted in SSA. We identified four full-length FFQ that have been validated for use in SSA populations: (i) a 164-item quantitative FFQ with a recall period of 7 d was validated against 2 d weighed food records using data from seventy participants in Mali; (ii) a 122-item FFQ was validated against four 24 h diet recalls using data from seventy-nine participants in Botswana; (iii) a 145-item FFQ was validated against a 7 d weighed food record using data from seventy-four participants in South Africa; and (iv) a quantitative FFQ was validated against two 24 h diet recalls using data from fifty women in Tanzania (information was not provided on number of food items)^(^ ^25^ ^,^ ^27^ ^,^ ^29^ ^,^ ^30^ ^)^. We found a median correlation of 0·35 for food groups, which is comparable to median correlations of 0·28, 0·8 and 0·37 that were found in Mali, Botswana and South Africa, respectively. Our food group correlations ranged from 0·00 to 0·51, compared with correlations ranging from −0·04 to 0·56, 0·18 to 0·58 and 0·14 to 0·56 in Mali, Botswana and South Africa, respectively.

For most nutrients and food groups that we examined, we found relatively similar population-level intakes to what has been found by other researchers in Tanzania. Lukmanji *et al*. found a similar distribution of macronutrient intakes in a study of pregnant women with HIV in Dar es Salaam^(^ ^33^ ^)^. A global study estimated that sugar-sweetened beverage intake in Tanzanians aged 40 years or older ranged from 0·16 to 0·32 servings/d, depending on age and sex^(^ ^49^ ^)^. This is similar in range to our findings of 0·2 servings/d, according to the FFQ, and 0·1 servings/d, according to the 24 h diet recalls. However, we also observed some intake values that differed considerably from previous studies. For example, a global study estimated that, on average, Tanzanians consume 2·75 (95 % CI 2·45, 3·08) g Na/d^(^ ^50^ ^)^, which is more than double the intake of 0·96 g/d estimated from our FFQ and 0·38 g/d estimated from our 24 h diet recalls.

Our study had several limitations. We used two 24 h diet recalls as our reference method instead of several weighed food records. Our 24 h diet recalls may have underestimated dietary intake due to multiple factors including under-reporting due to recall bias (e.g. forgot to report snacks or small food items) and under-representativeness of weekend diet (which is often larger than weekday diet). Other studies have found that participants incorrectly report portion sizes on 24 h diet recalls^(^ ^51^ ^)^ and a meta-analysis of FFQ validation studies found that correlations of nutrient intakes were lower for FFQ validated against 24 h diet recalls rather than food records^(^ ^52^ ^)^. However, weighed food records may affect participants’ behaviour and it is not possible to conduct food records in populations that have low literacy levels such as ours, unless an interviewer observes and records every meal, as was done for the FFQ validation in Mali^(^ ^25^ ^)^. The meta-analysis of FFQ validation studies also found that nutrient correlations were lower when the reference method of dietary questionnaire was conducted fewer than eight times^(^ ^52^ ^)^. We conducted two 24 h diet recalls per participant due to financial and logistic constraints. Further factors that may have lowered the agreement and correlation between our FFQ and the reference method were the limited training received by the study interviewers in how to conduct dietary questionnaires (i.e. one full-day session and two days of field tests) and questionnaire fatigue, for both participants and interviewers, due to the lengthy questionnaires conducted prior to the dietary questionnaires. The non-dietary DUCS-HTN questionnaire and measurements took approximately 1 h to complete and each dietary questionnaire took 30–45 min to complete.

Our FFQ queried participants about many individual foods. A future study could include questions on frequency of consumption of food groups in addition to questions on individual food items. This may help quantify whether asking about individual foods overestimates the food group consumption. It is possible that an FFQ that focused on the most common mixed dishes and amount and types of sauces added to dishes, rather than asking mainly about individual food items, might improve dietary assessment in this population.

In summary, our results indicate moderate agreement between the FFQ and two 24 h diet recalls for use in urban Tanzania. Our results could be used to inform the creation and implementation of improved dietary questionnaires for use in SSA. Future FFQ validation studies may wish to examine the effect of intensity of interviewer training on FFQ validation quality. In addition, future studies might conduct FFQ validations using more than two 24 h diet recalls spaced out over a longer period of time as the reference method.

## Supplementary material

For supplementary material accompanying this paper visit https://doi.org/10.1017/S1368980018000848. click here to view supplementary material
