# Supplementary material for: Validity of an FFQ to measure nutrient and food intakes in Tanzania
Source: Public Health Nutr. 2018 Apr 16;21(12):2211–20. doi: 10.1017/S1368980018000848 (PMC6101256; doi:10.1017/S1368980018000848)
Supplement: Supplementary file 1 [file S1368980018000848sup001.docx]

**Online Supplementary Material**

**Validity of a Food Frequency Questionnaire to Measure Nutrient and Food Intake in Tanzania**

***Page Content***

2 Composition of food groups from food frequency questionnaire (FFQ) food items in the Dar es Salaam Urban Cohort Hypertension Study (DUCS-HTN), 2014

3 Portion size images used by interviewers to conduct 24-hour diet recalls and FFQs

8 Dar es Salaam Urban Cohort Hypertension Study (DUCS-HTN) food frequency questionnaire

**Supplemental Table 1**. Composition of food groups from food frequency questionnaire (FFQ) food items in the Dar es Salaam Urban Cohort Hypertension Study (DUCS-HTN), 2014

| **Foods or food groups** | **Food items from the Food Frequency Questionnaire (FFQ)** |
| --- | --- |
| Cereals | Oat porridge or oatmeal, wheat porridge, millet meal porridge, corn porridge, rice porridge, millet porridge or stiff porridge (ugali), porridge with grain from factory, grain from factory, maize on cob, maize meal cooked as in stiff porridge (ugali), corn/sugar derived from corn juice, maize cooked with beans (kande), maize cooked with beans (kande) and rice, pilau rice, plain boiled rice, pasta, bread, scones, chapatti, pancakes, donut, rice cakes |
| Root vegetables | Cassava porridge, potato boiled or baked, cassava boiled, cassava stiff porridge (ugali), cassava in a mixed dish, sweet potato alone, sweet potato in a mixed dish, yam boiled alone, yam fried, yam in a mixed dish, pounded yam or other root vegetable |
| Legumes & nuts | Beans/legumes alone, beans in mixed dish, bean soup, bean cakes, bambara nuts, groundnuts alone, groundnuts with food, cashew nuts |
| Unprocessed red meat | Beef not minced, beef minced, goat, pork, lamb, offal, liver, meat samosa |
| Chicken | Chicken |
| Eggs | Eggs |
| Fish | Dried fish, anchovies (dagaa), canned tuna in salt water, canned tuna in oil, canned fish in salt water, canned fish in tomato broth, fish curry, fresh fish |
| Dairy | Powdered whole fat cows milk, powdered fat free cows milk, cow’s milk full fat, cow’s milk low fat, cow’s milk fat-free, non-dairy creamer, plain yogurt, artificially sweetened yogurt, ice cream, cheese soft but not in liquid, cheese in liquid, other types of cheese, ghee, butter |
| Fruit | Ripe banana, mango, tamarind, plum, papaya, tangerine, lemon or lime, jackfruit, baobab, watermelon, guava, peaches, pineapple, passion fruit, orange, apple, grapes, apricot, pears, sweet melon, blood fruit (matunda damu), berries, fruit canned in syrup, raisins and other dried fruit, avocado |
| Vegetables | Cucumber, spinach, lettuce, cabbage, amaranth leaves, pumpkin leaves, cow pea leaves, cassava leaves, chinese cabbage, other cabbage, other green leafy vegetables, pumpkin, okra alone, okra in mixed dish, green peas alone, green peas in mixed dish, tomato fresh not in salad, tomato fresh in salad, tomato cooked, carrot fresh not in salad, green pepper fresh not in salad, green pepper fresh as in salad, vegetable samosa, zucchini, green beans, beet, broccoli, cauliflower, eggplant alone, eggplant in mixed dish, mushrooms, onions |
| Sugar-sweetened beverages | Soda, squash or syrup |
| Alcohol | Fermented mealie pap drink, beer commercially prepared, beer locally brewer or homemade, wine commercially prepared, wine homemade or locally brewed, distilled alcoholic beverages commercially prepared, distilled alcoholic beverages |
| Tea | Tea with milk, tea without milk |

**Portion size images used by interviewers to conduct 24-hour diet recalls and FFQs**

**
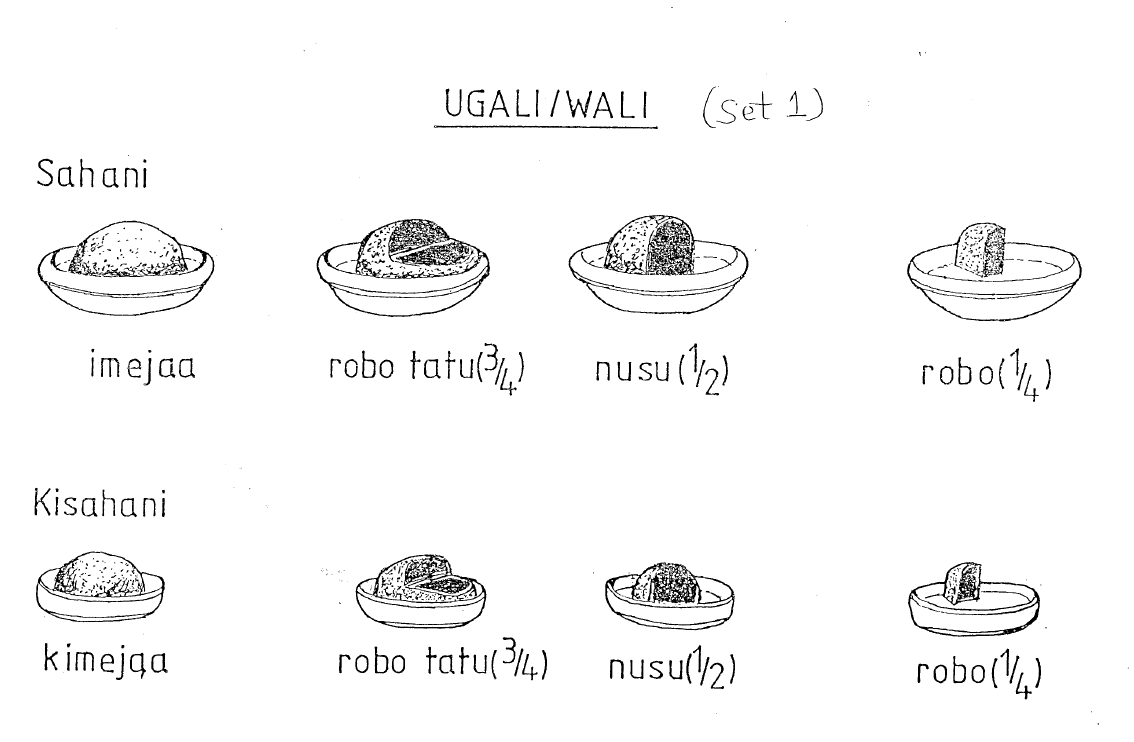
**

**
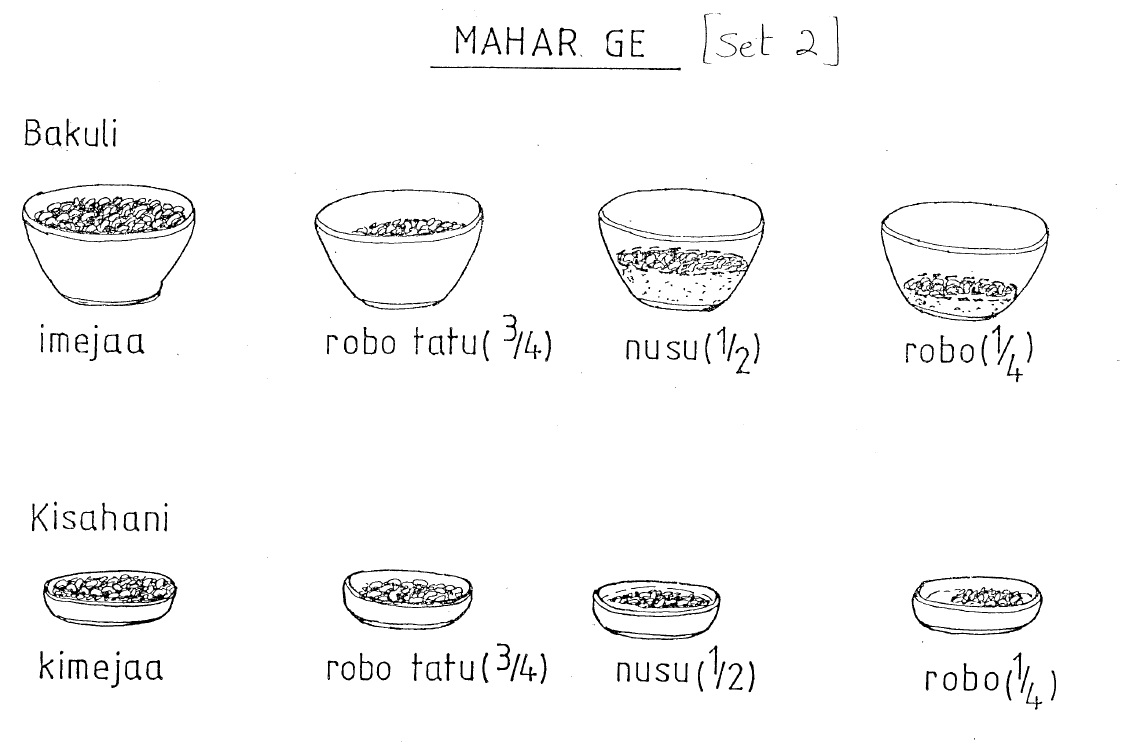
**

**
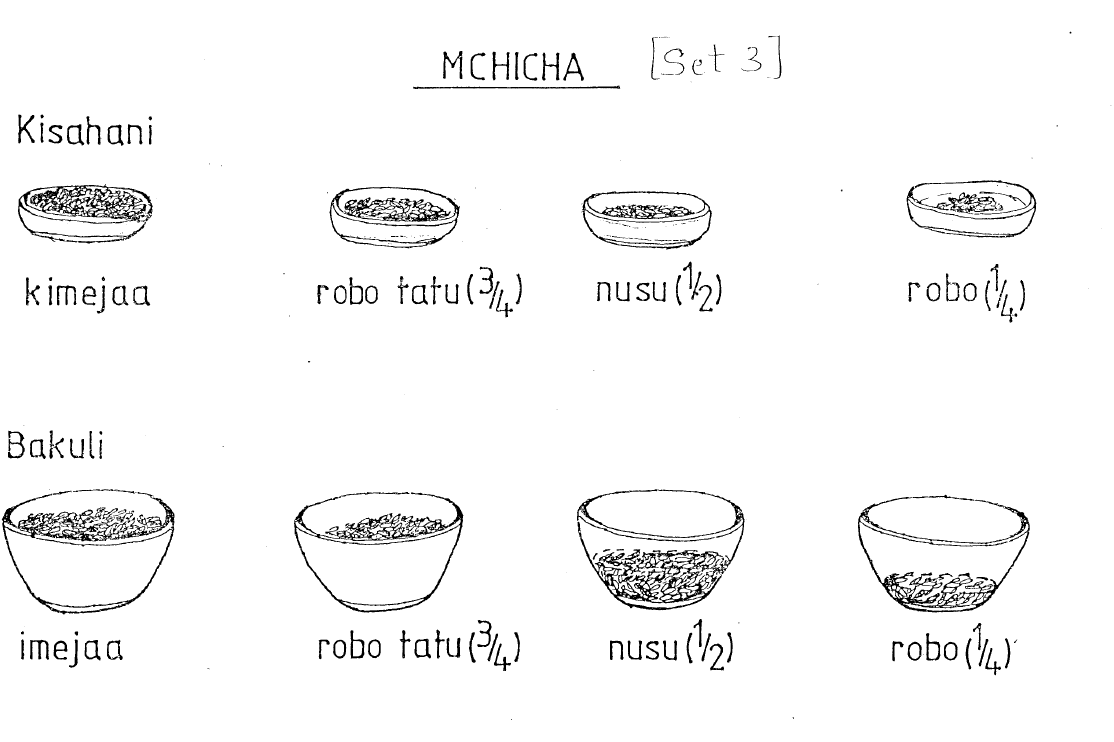
**

**
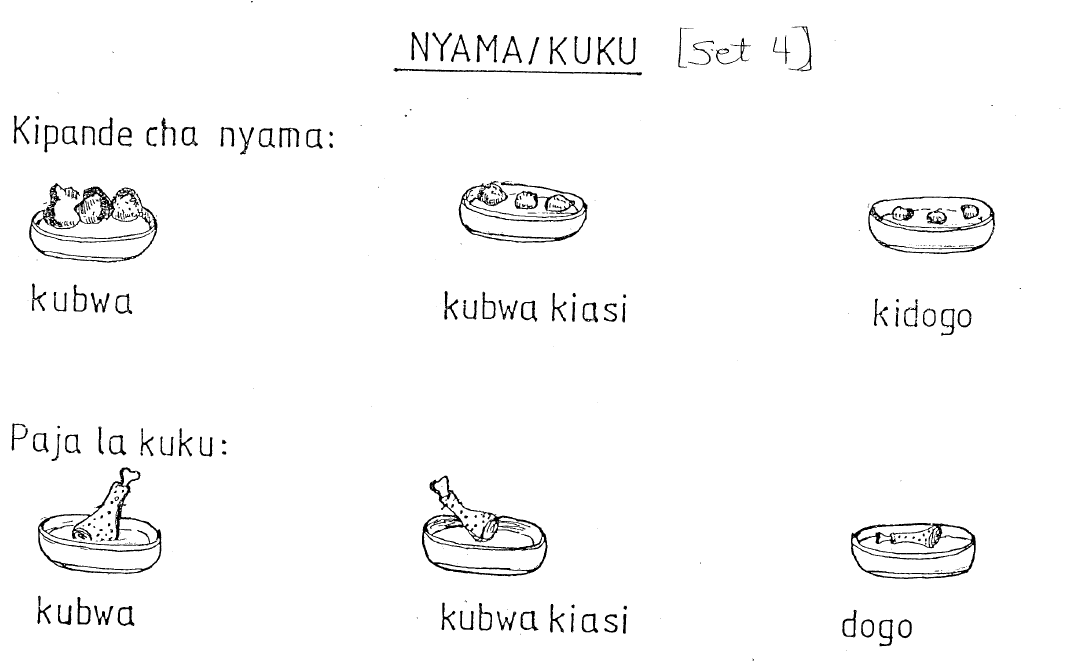
**

**
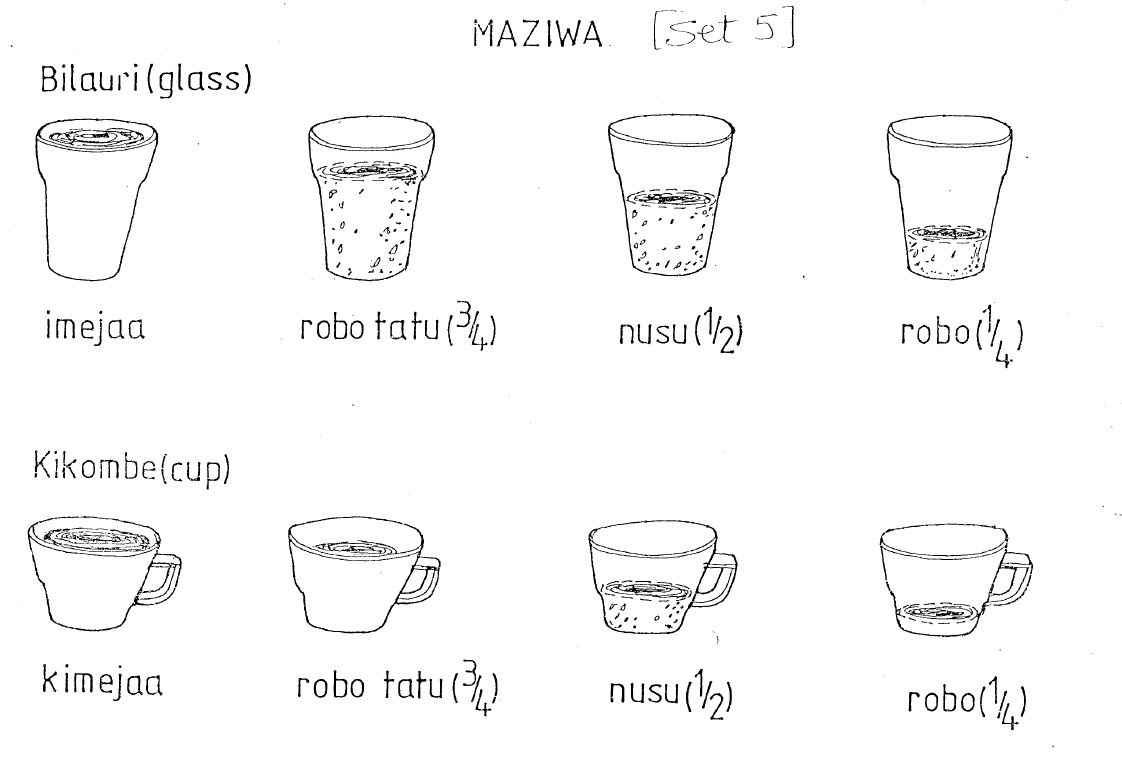
Dar es Salaam Urban Cohort Hypertension Study (DUCS-HTN) Food Frequency Questionnaire**

**How often have you eaten the following foods in the past 30 days?**

Do not count periods of fasting, such as Ramadan. Please pick only one frequency for each food. A note about serving sizes: 1 serving spoon = ½ cup. If you eat 2 servings of a food once a week, then you will check “2-4 per week.” If participants eat using handfuls from a shared container, change the serving size to number of handfuls. For the seasonal fruits and vegetable column, check the column if the participant eats the food only when it is in season.

**Grain, Potatoes, and Related Foods**

| Code | Food | Serving  Size | Never | 1-3 per month | 1  per week | 2-4 per week | 5-6 per week | 1 per day | 2-3 per day | 4-5 per day | 6+ per day |
| --- | --- | --- | --- | --- | --- | --- | --- | --- | --- | --- | --- |
| FFQ001 | Oat porridge or oatmeal (uji) | 1 cup |  |  |  |  |  |  |  |  |  |
| FFQ002 | Wheat porridge (uji) | 1 cup |  |  |  |  |  |  |  |  |  |
| FFQ003 | Millet meal porridge (uji) | 1 cup |  |  |  |  |  |  |  |  |  |
| FFQ004 | Corn porridge (uji) | 1 cup |  |  |  |  |  |  |  |  |  |
| FFQ005 | Cassava porridge (uji) | 1 cup |  |  |  |  |  |  |  |  |  |
| FFQ006 | Rice porridge (uji) | 1 cup |  |  |  |  |  |  |  |  |  |
| FFQ007 | Sorghum stiff porridge (ugali) | 1 cup |  |  |  |  |  |  |  |  |  |
| FFQ008 | Millet porridge or stiff porridge (uji or ugali) | 1 cup |  |  |  |  |  |  |  |  |  |
| FFQ009 | Pearl millet stiff porridge (ugali) | 1 cup |  |  |  |  |  |  |  |  |  |
| FFQ010 | Mixed grain porridge (uji) | 1 cup |  |  |  |  |  |  |  |  |  |
| FFQ011 | Grain from factory | 1 cup |  |  |  |  |  |  |  |  |  |
| Code | Food | Serving  Size | Never | 1-3 per month | 1  per week | 2-4 per week | 5-6 per week | 1 per day | 2-3 per day | 4-5 per day | 6+ per day |
| FFQ012 | Maize (roasted or boiled, on the cob) | 1 whole cob |  |  |  |  |  |  |  |  |  |
| FFQ013 | Maize meal, cooked, as in stiff porridge (ugali) | 1 plate |  |  |  |  |  |  |  |  |  |
| FFQ014 | Corn / sugar derived from corn juice | ½ cup |  |  |  |  |  |  |  |  |  |
| FFQ015 | Maize cooked with beans (Kande) | 1 plate |  |  |  |  |  |  |  |  |  |
| FFQ016 | Maize cooked with beans (Kande) and rice | 1 plate |  |  |  |  |  |  |  |  |  |
| FFQ017 | Rice, mixed as in a pilau, fried | 1 plate |  |  |  |  |  |  |  |  |  |
| FFQ018 | Plain rice, boiled | 1 plate |  |  |  |  |  |  |  |  |  |
| FFQ019 | Potato, boiled or baked | 1 potato, fist-sized |  |  |  |  |  |  |  |  |  |
| FFQ020 | Cassava, boiled (alone) | 1 medium size piece |  |  |  |  |  |  |  |  |  |
| FFQ021 | Cassava stiff porridge (ugali) | 1 piece, fist-sized |  |  |  |  |  |  |  |  |  |
| FFQ022 | Cassava (in a mixed dish) | 1 plate |  |  |  |  |  |  |  |  |  |
| FFQ023 | Sweet potato (alone) | 1 medium size |  |  |  |  |  |  |  |  |  |
| FFQ024 | Sweet potato (in a mixed dish) | 1 plate |  |  |  |  |  |  |  |  |  |
| FFQ025 | Taro (alone) | 1 piece, fist-sized |  |  |  |  |  |  |  |  |  |
| FFQ026 | Taro (in a mixed dish) | 1 plate |  |  |  |  |  |  |  |  |  |
| Code | Food | Serving  Size | Never | 1-3 per month | 1  per week | 2-4 per week | 5-6 per week | 1 per day | 2-3 per day | 4-5 per day | 6+ per day |
| FFQ027 | Yam (boiled, alone) | 1 piece, fist-sized |  |  |  |  |  |  |  |  |  |
| FFQ028 | Yam (fried) | 1 piece, fist-sized |  |  |  |  |  |  |  |  |  |
| FFQ029 | Yam (in a mixed dish) | 1 plate |  |  |  |  |  |  |  |  |  |
| FFQ030 | Pounded Yam or other root vegetable | 1 serving, fist-sized |  |  |  |  |  |  |  |  |  |
| FFQ031 | Plantain (boiled or steamed) (Ndizi) | 1 banana finger |  |  |  |  |  |  |  |  |  |
| FFQ032 | Plantain (roasted) (Ndizi) | 1 banana finger |  |  |  |  |  |  |  |  |  |
| FFQ033 | Plantain (fried, or chips) (Ndizi) | 1 banana finger |  |  |  |  |  |  |  |  |  |
| FFQ034 | Plantain (in a mixed dish) (Ndizi) | 1 plate |  |  |  |  |  |  |  |  |  |
| FFQ035 | Pasta, cooked (spaghetti, macaroni) | 1 plate |  |  |  |  |  |  |  |  |  |
| FFQ036 | Bread, breadrolls, buns | 1 slice/roll |  |  |  |  |  |  |  |  |  |
| FFQ037 | Scones, muffins | 1 medium size |  |  |  |  |  |  |  |  |  |
| FFQ038 | Flat bread (Chapati) | 1 round piece |  |  |  |  |  |  |  |  |  |
| FFQ039 | Pancakes (Chapati ya maji) | 1 piece |  |  |  |  |  |  |  |  |  |
| FFQ040 | Donut, fried dough (maandazi, kalimati, half-cake) | 1 piece |  |  |  |  |  |  |  |  |  |
| FFQ041 | Rice cakes (mkate kumimina) | 2 pieces |  |  |  |  |  |  |  |  |  |
| Code | Food | Serving  Size | Never | 1-3 per month | 1  per week | 2-4 per week | 5-6 per week | 1 per day | 2-3 per day | 4-5 per day | 6+ per day |
| FFQ042 | Cookies (biscuits) | 2 pieces |  |  |  |  |  |  |  |  |  |
| FFQ043 | Cake | 1 medium slice |  |  |  |  |  |  |  |  |  |

**Fruits**

| Code | Food | Serving  size | Never | 1-3 per month | 1  per week | 2-4 per week | 5-6 per week | 1 per day | 2-3 per day | 4-5 per day | 6+ per day |
| --- | --- | --- | --- | --- | --- | --- | --- | --- | --- | --- | --- |
| FFQ044 | Ripe banana | 1 banana finger |  |  |  |  |  |  |  |  |  |
| FFQ045 | Mango | 1 medium fruit |  |  |  |  |  |  |  |  |  |
| FFQ046 | Tamarind | 1 medium fruit |  |  |  |  |  |  |  |  |  |
| FFQ047 | Plum | 1 medium fruit |  |  |  |  |  |  |  |  |  |
| FFQ048 | Papaya | 1 slice |  |  |  |  |  |  |  |  |  |
| FFQ049 | Tangerine | 1 medium fruit |  |  |  |  |  |  |  |  |  |
| FFQ050 | Lemon or lime | 1 medium fruit |  |  |  |  |  |  |  |  |  |
| FFQ051 | Jackfruit | 1 finger piece |  |  |  |  |  |  |  |  |  |
| FFQ052 | Baobab | 1 handful |  |  |  |  |  |  |  |  |  |
| FFQ053 | Watermelon | 1 slice |  |  |  |  |  |  |  |  |  |
| FFQ054 | Guava | 1 medium fruit |  |  |  |  |  |  |  |  |  |
| FFQ055 | Peaches | 1 medium fruit |  |  |  |  |  |  |  |  |  |
| FFQ056 | Avocado | ½ medium fruit |  |  |  |  |  |  |  |  |  |
| FFQ057 | Pineapple (fruit only, not juice) | 1 thick slice |  |  |  |  |  |  |  |  |  |
| Code | Food | Serving  size | Never | 1-3 per month | 1  per week | 2-4 per week | 5-6 per week | 1 per day | 2-3 per day | 4-5 per day | 6+ per day |
| FFQ058 | Passion fruit (fruit only, not juice) | 1 medium fruit |  |  |  |  |  |  |  |  |  |
| FFQ059 | Orange (fruit only, not juice) | 1 medium fruit |  |  |  |  |  |  |  |  |  |
| FFQ060 | Orange juice | One 250 ml glass |  |  |  |  |  |  |  |  |  |
| FFQ061 | Other fruit juice | One 250 ml glass |  |  |  |  |  |  |  |  |  |
| FFQ062 | Apple | 1 medium fruit |  |  |  |  |  |  |  |  |  |
| FFQ063 | Grapes | 1 medium bunch |  |  |  |  |  |  |  |  |  |
| FFQ064 | Apricot | 3 medium |  |  |  |  |  |  |  |  |  |
| FFQ065 | Pears | 1 medium fruit |  |  |  |  |  |  |  |  |  |
| FFQ066 | Sweet melon | 1 slice |  |  |  |  |  |  |  |  |  |
| FFQ067 | Blood fruit (matunda damu) | 1 medium fruit |  |  |  |  |  |  |  |  |  |
| FFQ068 | Berries (strawberries, raspberries) | ½ cup |  |  |  |  |  |  |  |  |  |
| FFQ069 | Fruit canned in syrup | ½ cup |  |  |  |  |  |  |  |  |  |
| FFQ070 | Raisins, and other dried fruit | 1 handful |  |  |  |  |  |  |  |  |  |

**Legumes, Vegetables and Nuts:** Please note that 1 ladle or large serving spoon is equal to ½ cup

| Code | Food | Serving  size | Never | 1-3 per month | 1  per week | 2-4 per week | 5-6 per week | 1 per day | 2-3 per day | 4-5 per day | 6+ per day |
| --- | --- | --- | --- | --- | --- | --- | --- | --- | --- | --- | --- |
| FFQ071 | Beans/legumes alone (Examples: mung & soybeans; pigeon, cow & chick peas, split peas, lentils, dried beans) | ½ plate |  |  |  |  |  |  |  |  |  |
| FFQ072 | Beans in mixed dish | 1 plate |  |  |  |  |  |  |  |  |  |
| FFQ073 | Bean soup | 1 plate |  |  |  |  |  |  |  |  |  |
| FFQ074 | Bean cakes (Bhajia) | 1 |  |  |  |  |  |  |  |  |  |
| FFQ075 | Bambara nuts | ½ plate |  |  |  |  |  |  |  |  |  |
| FFQ076 | Groundnuts (alone) | ½ plate |  |  |  |  |  |  |  |  |  |
| FFQ077 | Groundnuts (added to food) | 1 plate |  |  |  |  |  |  |  |  |  |
| FFQ078 | Cashew nuts | ½ cup |  |  |  |  |  |  |  |  |  |
| FFQ079 | Cucumber | ½ plate |  |  |  |  |  |  |  |  |  |
| FFQ080 | Spinach, cooked | ½ plate |  |  |  |  |  |  |  |  |  |
| FFQ081 | Lettuce | 1 plate |  |  |  |  |  |  |  |  |  |
| FFQ082 | Cabbage | 1 plate |  |  |  |  |  |  |  |  |  |
| FFQ083 | Amaranth leaves, cooked | ½ cup |  |  |  |  |  |  |  |  |  |
| Code | Food | Serving  size | Never | 1-3 per month | 1  per week | 2-4 per week | 5-6 per week | 1 per day | 2-3 per day | 4-5 per day | 6+ per day |
| FFQ084 | Pumpkin leaves, cooked | ½ cup |  |  |  |  |  |  |  |  |  |
| FFQ085 | Cow pea leaves, cooked | ½ cup |  |  |  |  |  |  |  |  |  |
| FFQ086 | Cassava leaves | ½ cup |  |  |  |  |  |  |  |  |  |
| FFQ087 | Chinese cabbage, cooked | ½ cup |  |  |  |  |  |  |  |  |  |
| FFQ088 | Other cabbage, cooked | ½ cup |  |  |  |  |  |  |  |  |  |
| FFQ089 | Other green leafy vegetables, cooked | ½ cup |  |  |  |  |  |  |  |  |  |
| FFQ090 | Pumpkin, or Butternut, cooked | ½ plate |  |  |  |  |  |  |  |  |  |
| FFQ091 | Okra (alone) | ½ cup |  |  |  |  |  |  |  |  |  |
| FFQ092 | Okra (in a mixed dish) | 1 cup |  |  |  |  |  |  |  |  |  |
| FFQ093 | Green peas (alone) | ½ plate |  |  |  |  |  |  |  |  |  |
| FFQ094 | Green peas (in a mixed dish) | 1 plate |  |  |  |  |  |  |  |  |  |
| FFQ095 | Bitter tomato (alone) | ½ cup |  |  |  |  |  |  |  |  |  |
| FFQ096 | Bitter tomato (in a mixed dish) | 1 cup |  |  |  |  |  |  |  |  |  |
| FFQ097 | Tomato (fresh, not in salad) | 1 fruit |  |  |  |  |  |  |  |  |  |
| FFQ098 | Tomato (fresh, as in salad) | 2 slices |  |  |  |  |  |  |  |  |  |
| Code | Food | Serving  size | Never | 1-3 per month | 1  per week | 2-4 per week | 5-6 per week | 1 per day | 2-3 per day | 4-5 per day | 6+ per day |
| FFQ099 | Tomato, cooked | ½ cup |  |  |  |  |  |  |  |  |  |
| FFQ100 | Carrot (fresh, not in salad) | 1 carrot |  |  |  |  |  |  |  |  |  |
| FFQ101 | Carrot (fresh, as in salad) | ½ carrot |  |  |  |  |  |  |  |  |  |
| FFQ102 | Green pepper (fresh, not in salad) | ½ cup |  |  |  |  |  |  |  |  |  |
| FFQ103 | Green pepper (fresh, as in salad) | ¼ small |  |  |  |  |  |  |  |  |  |
| FFQ104 | Pie, pastry with vegetable filling (vegetable samosa) | 1 piece |  |  |  |  |  |  |  |  |  |
| FFQ105 | Zucchini, cooked | ½ cup |  |  |  |  |  |  |  |  |  |
| FFQ106 | Green beans, cooked | ½ cup |  |  |  |  |  |  |  |  |  |
| FFQ107 | Beetroot, cooked | ½ cup |  |  |  |  |  |  |  |  |  |
| FFQ108 | Broccoli, cooked | ½ cup |  |  |  |  |  |  |  |  |  |
| FFQ109 | Cauliflower, cooked | ½ cup |  |  |  |  |  |  |  |  |  |
| FFQ110 | Eggplant (alone), cooked | 1 slice |  |  |  |  |  |  |  |  |  |
| FFQ111 | Eggplant (in a mixed dish), cooked | ½ cup |  |  |  |  |  |  |  |  |  |
| FFQ112 | Mushrooms, cooked | ½ cup |  |  |  |  |  |  |  |  |  |
| FFQ113 | Onions (cooked with oil) | 1/2 cup |  |  |  |  |  |  |  |  |  |

**Meat, fish and eggs**

| Code | Food | Serving  Size | Never | 1-3 per month | 1  per week | 2-4 per week | 5-6 per week | 1 per day | 2-3 per day | 4-5 per day | 6+ per day |
| --- | --- | --- | --- | --- | --- | --- | --- | --- | --- | --- | --- |
| FFQ114 | Beef (not minced) | 1 palm-sized serving |  |  |  |  |  |  |  |  |  |
| FFQ115 | Beef mince | ½ cup |  |  |  |  |  |  |  |  |  |
| FFQ116 | Goat | 1 palm-sized serving |  |  |  |  |  |  |  |  |  |
| FFQ117 | Pork | 1 palm-sized serving |  |  |  |  |  |  |  |  |  |
| FFQ118 | Lamb/Mutton | 1 palm-sized serving |  |  |  |  |  |  |  |  |  |
| FFQ119 | Offal/Tripe | 1 palm-sized serving |  |  |  |  |  |  |  |  |  |
| FFQ120 | Liver | 1 palm-sized serving |  |  |  |  |  |  |  |  |  |
| FFQ121 | Chicken | 1 palm-sized serving |  |  |  |  |  |  |  |  |  |
| FFQ122 | Sausage | 1 piece (length of a hand) |  |  |  |  |  |  |  |  |  |
| FFQ123 | Bacon | 3-4 rashers |  |  |  |  |  |  |  |  |  |
| FFQ124 | Processed meat (bologna) | 2 pieces |  |  |  |  |  |  |  |  |  |
| FFQ125 | Processed meat (Vienna sausages) | 2 pieces |  |  |  |  |  |  |  |  |  |
| FFQ126 | Ham | 2 pieces |  |  |  |  |  |  |  |  |  |
| FFQ127 | Canned beef | ½ plate/bowl |  |  |  |  |  |  |  |  |  |
| Code | Food | Serving  Size | Never | 1-3 per month | 1  per week | 2-4 per week | 5-6 per week | 1 per day | 2-3 per day | 4-5 per day | 6+ per day |
| FFQ128 | Pie, pastry with meat filling (meat samosa) | 1 piece |  |  |  |  |  |  |  |  |  |
| FFQ129 | Dried fish | 1 palm-sized serving |  |  |  |  |  |  |  |  |  |
| FFQ130 | Anchovies/sardines (dagaa) | 4-5 sardines or ½ cup |  |  |  |  |  |  |  |  |  |
| FFQ131 | Canned tuna in salt water | ½ bowl |  |  |  |  |  |  |  |  |  |
| FFQ132 | Canned tuna in oil | ½ bowl |  |  |  |  |  |  |  |  |  |
| FFQ133 | Canned fish in salt water | ½ cup or two fish |  |  |  |  |  |  |  |  |  |
| FFQ134 | Canned fish in tomato water | ½ cup or two fish |  |  |  |  |  |  |  |  |  |
| FFQ135 | Fish curry/stew | ½ cup |  |  |  |  |  |  |  |  |  |
| FFQ136 | Fish, fresh  (Other forms of fish - not canned) | 1 palm-sized serving |  |  |  |  |  |  |  |  |  |
| FFQ137 | Eggs | 1 egg |  |  |  |  |  |  |  |  |  |

**Dairy Foods**

| Code | Food | Serving  Size | Never | 1-3 per month | 1  per week | 2-4 per week | 5-6 per week | 1 per day | 2-3 per day | 4-5 per day | 6+ per day |
| --- | --- | --- | --- | --- | --- | --- | --- | --- | --- | --- | --- |
| FFQ138 | Powdered cow’s milk, whole or full cream | One 250 ml glass |  |  |  |  |  |  |  |  |  |
| FFQ139 | Powdered cow’s milk, fat free or non-fat | One 250 ml glass |  |  |  |  |  |  |  |  |  |
| FFQ140 | Cow’s milk; full cream, whole, or direct from the cow | One 250 ml glass |  |  |  |  |  |  |  |  |  |
| FFQ141 | Cow’s milk; low fat or 2% | One 250 ml glass |  |  |  |  |  |  |  |  |  |
| FFQ142 | Cow’s milk; fat-free, or non-fat | One 250 ml glass |  |  |  |  |  |  |  |  |  |
| FFQ143 | Non-dairy creamer (Cremora) | 2 heaped teaspoons |  |  |  |  |  |  |  |  |  |
| FFQ144 | Yogurt, plain | 1 cup |  |  |  |  |  |  |  |  |  |
| FFQ145 | Yogurt, artificially sweetened | 1 cup |  |  |  |  |  |  |  |  |  |
| FFQ146 | Yogurt, sweetened | 1 cup |  |  |  |  |  |  |  |  |  |
| FFQ147 | Ice cream | 1 cup |  |  |  |  |  |  |  |  |  |
| FFQ148 | Cheese, soft but not in liquid | ½ cup |  |  |  |  |  |  |  |  |  |
| FFQ149 | Cheese, in liquid | 28 g or about the size of a small matchbox |  |  |  |  |  |  |  |  |  |
| FFQ150 | Other types of cheese, | 1 slice or 28 g or about the size of a small matchbox |  |  |  |  |  |  |  |  |  |

**Beverages**

| Code | Food | Serving  Size | Never | 1-3 per month | 1  per week | 2-4 per week | 5-6 per week | 1 per day | 2-3 per day | 4-5 per day | 6+ per day |
| --- | --- | --- | --- | --- | --- | --- | --- | --- | --- | --- | --- |
| FFQ151 | Tea with milk | 1 cup |  |  |  |  |  |  |  |  |  |
| FFQ152 | Tea without milk | 1 cup |  |  |  |  |  |  |  |  |  |
| FFQ153 | Coffee with milk | 1 cup |  |  |  |  |  |  |  |  |  |
| FFQ154 | Coffee without milk | 1 cup |  |  |  |  |  |  |  |  |  |
| FFQ155 | Soda (Coke, Fanta, etc) | One 350 ml bottle |  |  |  |  |  |  |  |  |  |
| FFQ156 | Diet soda | One 350 ml bottle |  |  |  |  |  |  |  |  |  |
| FFQ157 | Squash or syrup mixed with water (with sugar) | One 250 ml glass |  |  |  |  |  |  |  |  |  |
| FFQ158 | Squash or syrup mixed with water (with artificial sweetener) | One 250 ml glass |  |  |  |  |  |  |  |  |  |
| FFQ159 | Soya drink | 1 cup |  |  |  |  |  |  |  |  |  |
| FFQ160 | Fermented mealie pap drink (mageu, amahewu, amarhewu) | 1 cup |  |  |  |  |  |  |  |  |  |
| FFQ161 | Coconut milk | 1 cup |  |  |  |  |  |  |  |  |  |
| FFQ162 | Beer, commercially prepared | One 250 ml glass |  |  |  |  |  |  |  |  |  |
| FFQ163 | Beer, homemade or locally brewed | One 250 ml glass |  |  |  |  |  |  |  |  |  |
| FFQ164 | Wine, commercially prepared | One-half 250 ml glass |  |  |  |  |  |  |  |  |  |
| Code | Food | Serving  Size | Never | 1-3 per month | 1  per week | 2-4 per week | 5-6 per week | 1 per day | 2-3 per day | 4-5 per day | 6+ per day |
| FFQ165 | Wine, homemade or locally brewed | One 250 ml glass |  |  |  |  |  |  |  |  |  |
| FFQ166 | Distilled alcoholic beverages, commercially prepared (examples: whisky, rum, vodka, gin) | 1 drink or shot or tot |  |  |  |  |  |  |  |  |  |
| FFQ167 | Distilled alcoholic beverages, homemade or locally brewed | 1 drink or shot or tot |  |  |  |  |  |  |  |  |  |

**Other foods**

| Code | Food | Serving  size | Never | 1-3 per month | 1 per week | 2-4 per week | 5-6 per week | 1 per day | 2-3 per day | 4-5 per day | 6+ per day |
| --- | --- | --- | --- | --- | --- | --- | --- | --- | --- | --- | --- |
| FFQ168 | Sugar added to foods (include in tea & coffee) | 1 teaspoon |  |  |  |  |  |  |  |  |  |
| FFQ169 | Honey or jam | 1 teaspoon |  |  |  |  |  |  |  |  |  |
| FFQ170 | Peanut butter | 1 heaped tsp |  |  |  |  |  |  |  |  |  |
| FFQ171 | Mayonnaise or salad dressing | 1 heaped tsp |  |  |  |  |  |  |  |  |  |
| FFQ172 | Chocolate bar | 1 bar |  |  |  |  |  |  |  |  |  |
| FFQ173 | Other sweets or candy | 1 handful, or 4-5 pieces |  |  |  |  |  |  |  |  |  |
| FFQ174 | Small pieces of roasted corn (crisps) | 1 cup |  |  |  |  |  |  |  |  |  |
| FFQ175 | Potato, crisps, Cassava crisps  French fries (Irish potato) | 1 cup |  |  |  |  |  |  |  |  |  |
| FFQ176 | Ghee (samli) | 1 heaped tsp |  |  |  |  |  |  |  |  |  |
| FFQ177 | Hard (unmelted) butter | 1 heaped tsp |  |  |  |  |  |  |  |  |  |
| FFQ178 | Soft (melted) butter | 1 heaped tsp |  |  |  |  |  |  |  |  |  |
| FFQ179 | Animal fat | 1 heaped tsp |  |  |  |  |  |  |  |  |  |

**Vitamins**

**FFQ180:** Do you currently take multi-vitamin tablets?

- No
- Yes, 2 or less per week
- Yes, 3-5 per week
- Yes, 6-9 per week
- Yes, 10 or more per week

**FFQ181:** Not counting multi-vitamins, do you take any other vitamins or supplements on a regular basis?

- No
- Yes

**FFQ181a:**  If yes, which? _ _ _ _ _ _ _ _ _ _ _ _ _ _ _ _ _

**Eating Behaviors**

| Code | Food | Never | 1-3 per month | 1  per week | 2-4 per week | 5-6 per week | 1 per day | 2-3 per day | 4-5 per day | 6+ per day |
| --- | --- | --- | --- | --- | --- | --- | --- | --- | --- | --- |
| FFQ182 | How many times per day do you eat? |  |  |  |  |  |  |  |  |  |
| FFQ183 | How often do you eat food prepared away from home, for example in a restaurant, cafeteria, or from a street stall? |  |  |  |  |  |  |  |  |  |
| FFQ184 | How often do you eat food that has been fried, at home? |  |  |  |  |  |  |  |  |  |
| FFQ185 | How often do you eat food that has been fried, away from home? |  |  |  |  |  |  |  |  |  |
| FFQ186 | How often do you add salt to foods on your plate? |  |  |  |  |  |  |  |  |  |
| FFQ187 | How often do you (or your partner) add salt to foods during cooking? |  |  |  |  |  |  |  |  |  |
| FFQ188 | How often do you (or your partner) use Maggi cubes, Aromat, Soy sauce, Vegemite when cooking at home? |  |  |  |  |  |  |  |  |  |
| FFQ189 | How often do you eat food rich in salt such as salted fish (Nguru), salted meat (Mishikaki), salami, salted peanuts, pizza, etc)? |  |  |  |  |  |  |  |  |  |

**FFQ190**: Do you think that a high salt intake can cause serious health problems?

- Yes
- No
- Refused
- Don’t know

**FFQ191**: If yes, can you mention one or two health problems that are related to high salt intake (if the participant doesn’t know, write “don’t know”):

_ _ _ _ _ _ _ _ _ _ _ _ _ _ _ _ _ _ _ _ _ _ _ _ _ _ _ _ _ _ _ _

_ _ _ _ _ _ _ _ _ _ _ _ _ _ _ _ _ _ _ _ _ _ _ _ _ _ _ _ _ _ _ _

**FFQ192**: Is it important for you to limit salt in your diet?

- Not really
- Somewhat important
- Really important
- Refused
- Don’t know

**FFQ193**: Do you do anything to limit your salt intake, e.g. avoid salty products or avoid adding salt in your food?

- Yes, often
- Yes, sometimes
- Not really
- Refused
- Don’t know

**FFQ194**: If yes, what do you do to control your salt intake?

_ _ _ _ _ _ _ _ _ _ _ _ _ _ _ _ _ _ _ __ _ _ _ _ _ _ _ _ _ _ _

_ _ _ _ _ _ _ _ _ _ _ _ _ _ _ _ _ _ _ __ _ _ _ _ _ _ _ _ _ _ _

**Cooking Oil**

**FFQ195**: **What is the main cooking oil used at home for frying? (Choose one)**

- Butter
- Margarine
- Ghee
- Olive oil
- Sunflower oil (SA: “fish oil”
- Groundnut oil
- Corn oil
- Soy oil
- Other vegetable oil (Korie)
- Other (specify) _ _ _ _ _ _ _ _ _ _ _ _
- Do not use oil
